# TXNRD1 Is an Unfavorable Prognostic Factor for Patients with Hepatocellular Carcinoma

**DOI:** 10.1155/2017/4698167

**Published:** 2017-04-27

**Authors:** Binsheng Fu, Wei Meng, Xiancheng Zeng, Hui Zhao, Wei Liu, Tong Zhang

**Affiliations:** ^1^Department of Hepatic Surgery and Liver Transplantation Center of the Third Affiliated Hospital, Organ Transplantation Institute of Sun Yat-sen University, Organ Transplantation Research Center of Guangdong Province, Guangzhou 510630, China; ^2^Department of General Surgery, The Second People's Hospital of Guangdong Province, Guangzhou 510317, China; ^3^Guangdong Provincial Key Laboratory of Liver Disease Research and Key Laboratory of Clinical & Translational Research on Biotherapy for Liver Disease of Guangdong Higher Education Institutes and Cell-Gene Therapy Translational Medicine Research Center of The Third Affiliated Hospital, Sun Yat-sen University, Guangzhou, Guangdong 510630, China

## Abstract

Thioredoxin reductase 1 (TXNRD1) which is a selenocysteine-containing protein is overexpressed in many malignancies. Its role in the hepatocellular carcinoma (HCC) prognosis has not been investigated. In this study, we investigated whether TXNRD1 functions as an independent prognostic factor for HCC patients. We found TXNRD1 was overexpressed in HCC tissues and cells, immunohistochemical analysis suggested TXNRD1 was elevated in 57 of 120 (47.5%) clinical samples, and its level was increased with the increasing clinical stage. In addition, TXNRD1 expression was positively correlated with clinical stage (*p* = 3.5*e* − 5), N classification (*p* = 4.4*e* − 4), and M classification (*p* = 0.037) of HCC patients. Kaplan-Meier analysis revealed that patients with high TXNRD1 expression had significantly shorter survival time than patients with low TXNRD1 expression. Multivariate analysis found TXNRD1 was an independent prognostic factor for HCC patients. In conclusion, our data suggested that TXNRD1 was a biomarker for the prognosis of patients with HCC.

## 1. Introduction

HCC is one of the most common human tumors worldwide. According to the data published by the World Cancer Report 2014, the global incidence and mortality of HCC are 6% and 9%, respectively. About 746,000 patients died at 2012. Despite the advances in diagnosis and therapy, HCC still has poor prognosis and high recurrence rate, and looking for new diagnostic factors and therapeutic factors is critical for HCC therapy [1].

Selenoprotein TXNRD1 is a critical antioxidant enzyme catalyzing the NADPH-dependent reduction of thioredoxin to regulate cellular redox homeostasis [2]. It associated with cell proliferation, apoptosis, and transformation [3]. TXNRD1 is also upregulated in many human malignancies and functions as a prognostic factor for many tumors, such as oral squamous cell carcinomas [4], lung cancer [5, 6], breast cancer [7, 8], and astrocytomas [9]. Previous reports have shown that the hepatocarcinogenesis rate in TXNRD1 knockout mice was significantly increased, induced by carcinogen diethylnitrosamine (DEN), suggesting it could protect against chemically induced hepatocarcinogenesis through controlling the balance of cellular redox state, but it could not induce hepatocarcinogenesis [10]. Anticancer natural product gambogic acid interacts with TXNRD1 to inhibit its Trx-reduction activity and to increase the levels of reactive oxygen species to induce apoptosis in human HCC cell SMMC-7721; meanwhile, knockdown of TXNRD1 attenuates the gambogic acid cytotoxicity [11]. These findings suggested TXNRD1 might regulate the progression of HCC, but whether TXNRD1 could serve as a novel prognostic factor for HCC patients has not been studied until now. In this study, we determined the role of TXNRD1 in the prognosis for HCC patients, and we showed that TXNRD1 was upregulated in HCC cells and tissues and was an unfavorable prognostic factor for HCC patients.

## 2. Materials and Methods

### 2.1. Cell Culture and Clinical Samples

Hepatocyte cell line LO2 cells were grown in RPMI-1640 medium (Gibco) supplemented with 10% fetal bovine serum (FBS, Gibco), 0.3% glutamine, 0.1 mM nonessential amino acids, 1 mM sodium pyruvate, and 10 *μ*M *β*-mercaptoethanol (Gibco). HCC cell lines including Hep3B, BEL-7404, HepG2, Huh7, QGY-7703, MHCC97H, MHCC97L, BEL-7402, and HCCC-9801 were maintained in DMEM medium (Hyclone) supplemented with 10% FBS (Gibco). These cells were obtained from ATCC.

A cohort of 120 paraffin-embedded HCC samples was obtained from the Third Affiliated Hospital of Sun Yat-sen University (Guangzhou, China) with informed consent according to the Institute Research Ethics Committee; these patients were histologically and clinically diagnosed between July 2008 and November 2014. The detailed information was shown in Table 1, clinicopathological characteristics such as gender, age, clinical stage, and TNM classification were included.

### 2.2. Western Blot

Cell lysates were extracted from cells using RIPA buffer (50 mM Tris (pH 7.4), 150 mM NaCl, 1% Triton X-100, 1% sodium deoxycholate, and 0.1% SDS supplemented with protease inhibitor cocktail (Roche)). Protein concentration was measured using BCA Protein Assay Kit (Pierce). Equivalent protein lysates were separated on 12% SDS-PAGE gels and transferred to PVDG membranes (Millipore) followed with blocking with 5% nonfat milk for 1 h at room temperature. After blocking, membranes were incubated with anti-TXNRD1 antibodies (1 : 1000, #15140, Cell Signaling) for 2 h at room temperature; then, the membranes were washed using TBST buffer and incubated with an anti-rabbit IgG, HRP-linked antibody (1 : 10000, #7074, Cell Signaling) for 1 h at room temperature. The band was detected using Amersham ECL Prime (GE Healthcare). The membranes were stripped and reprobed with an anti-*β*-actin antibody as the loading control.

### 2.3. Immunohistochemistry (IHC)

IHC was performed according to the standard method described previously [12, 13]. Two observers who were blinded to the clinical outcome evaluated and scored the degree of immunostaining of TXNRD1 independently. The staining index was calculated as the sum of the intensity of staining and the percentage of positively stained tumor cells. Scores for the intensity of staining were shown as follows: 0 (no staining), 1 (weak staining, light yellow), 2 (moderate staining, yellow brown), and 3 (strong staining, brown). Scores for the percentage of positively stained tumor cells were shown as follows: 0 (no positive tumor cells), 1 (<10% of the cells), 2 (10–50% of the cells), 3 (>50% of the cells). The staining index scores were 0, 1, 2, 3, 4, 6 and 9. The cut-off values for TXNRD1 expression were chosen based on the measure of heterogeneity using the log-rank test with respect to the overall survival. When SI score was ≥4, the tumor was considered to have high expression; otherwise, the tumor was considered to have low expression.

### 2.4. Statistical Analysis

All statistical analyses in this study were carried out using SPSS 13.0 (IBM). Chi-square test and Fisher's test were used to determine the relationship between TXNRD1 expression and the clinicopathological characteristics, and Spearman correlation test was performed to calculate bivariate correlations between the study variables. The survival curves were plotted by the Kaplan-Meier method and compared by log-rank test. Univariate and multivariate analysis was carried out using Cox's proportional hazards regression models. For all tests, a two-sided *p* value of less than 0.05 was considered to be statistically significant.

## 3. Results

### 3.1. TXNRD1 Is Overexpressed in HCC Tissues and Cells

**Figure 1:**
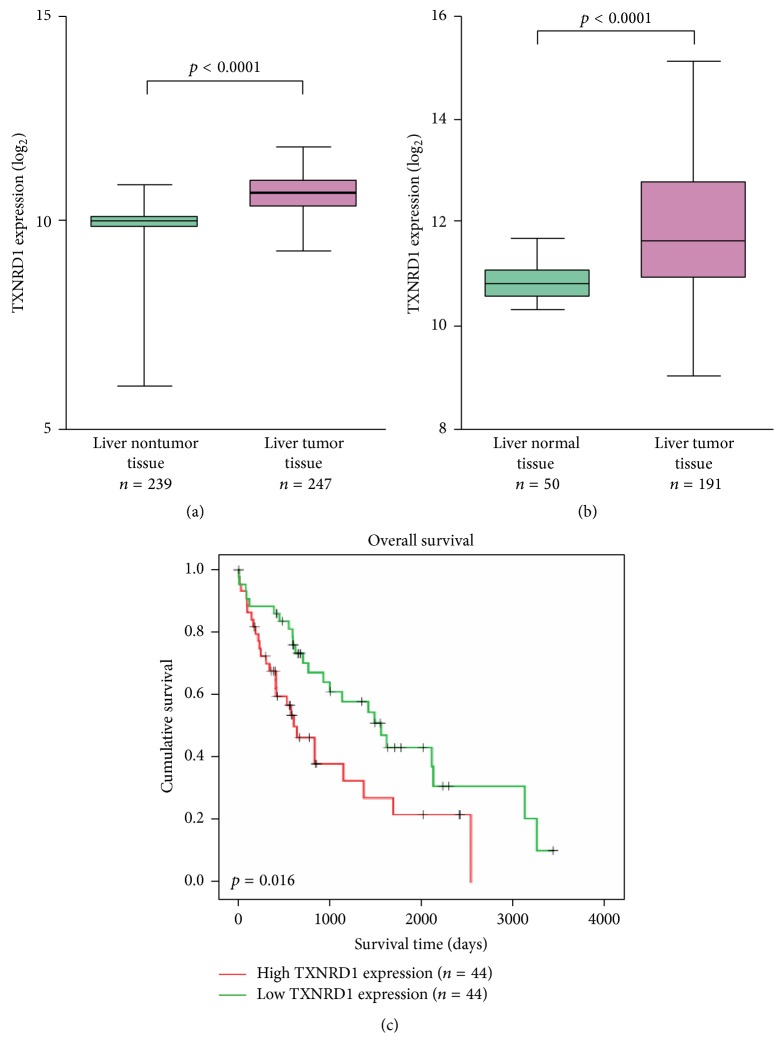
TXNRD1 was overexpressed in HCC tissues and patients with high TXNRD1 level had poor outcome. (a) The mRNA levels of TXNRD1 in HCC tissues and normal liver tissues, the data downloaded from GSE14520. (b) The mRNA levels of TXNRD1 in HCC tissues and normal liver tissues, the data downloaded from TCGA dataset. (c) Kaplan-Meier analysis for overall survival of HCC patients according to the level of TXNRD1. Data represent mean ± SD.

**Figure 2:**
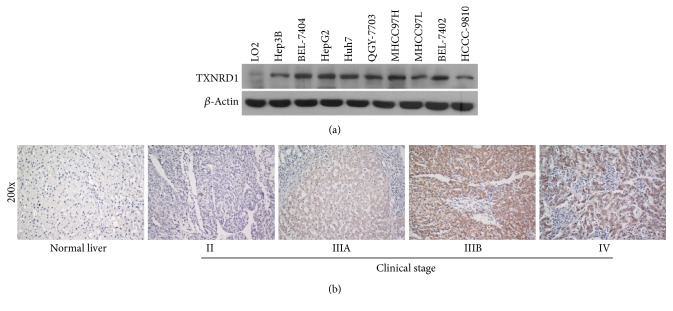
TXNRD1 expression was upregulated in HCC cells and was positively correlated with clinical stage. (a) Western blot analysis of TXNRD1 level in HCC cells and normal liver cell LO2. *β*-Actin was used as the loading control. (b) Representative photographs of TXNRD1 level in normal liver tissues, HCC samples in clinical stages II, IIIA, IIIB, and IV determined by IHC analysis. Original magnification: ×200.

To investigate the prognostic value of TXNRD1, we first examined TXNRD1 expression in HCC tissues. We found TXNRD1 mRNA level was significantly upregulated in HCC tissues compared to liver nontumor tissues (*p* < 0.0001), mRNA expression profiles of HCC and liver nontumor tissues came from GSE14520 (Figure 1(a)), we also used The Cancer Genome Atlas (TCGA) dataset to analyze TXNRD1 expression, and TXNRD1 mRNA level was also significantly upregulated in HCC tissues compared to normal live tissues (*p* < 0.0001) (Figure 1(b)). Kaplan-Meier survival curves suggested patients with high TXNRD1 have poor outcome (*p* = 0.016) (Figure 1(c)); this data also came from TCGA. Moreover, we determined TXNRD1 expression in HCC cells, and western blot assay suggested TXNRD1 protein level was upregulated in HCC cells compared to normal live cell LO2 (Figure 2(a)).

We further examined TXNRD1 protein level in 120 paraffin-embedded archived HCC tissues using IHC. IHC analysis suggested 113 (94.2%) samples expressed TXNRD1 positively, and only 7 (5.8%) samples expressed TXNRD1 negatively. 63 samples (52.5%) had low TXNRD1 expression, 57 (47.5%) had high TXNRD1 expression (Table 2), and TXNRD1 was mainly located in the cytoplasm. These samples included 30 stage I samples, 30 stage II samples, 34 stage III samples, and 26 stage IV samples. IHC assay also showed TXNRD1 level was positively correlated with advancing clinical stage (Figure 2(b)); the detailed data was shown as follows: 23% (7/30) for stage I, 37% (11/30) for stage II, 53% (18/34) for stage III, and 81% (21/27) for stage IV (Table 3). This result suggested high TXNRD1 expression was associated with advancing clinical stage. These results suggested TXNRD1 was overexpressed in HCC tissues and might be a poor prognostic factor.

### 3.2. Relationship between Clinicopathologic Features and TXNRD1 Expression in HCC

We investigated the relationship between TXNRD1 level and clinicopathological characteristics of HCC patients and found that TXNRD1 level was significantly correlated with clinical stage (*p* = 3.5*e* − 5) and N classification (*p* = 4.4*e* − 4 using Chi-square test, *p* = 0.001 using Fisher's exact test). TXNRD1 also was significantly correlated with M classification analyzed by Chi-square test (*p* = 0.037) but was not significantly correlated with M classification analyzed by Fisher's exact test (*p* = 0.052). However, TXNRD1 level does not have significant correlation with gender, age, or T classification (Table 3). We used Spearman correlation analysis to confirm this result; Spearman correlation analysis showed TXNRD1 level was significantly correlated with clinical stage (*r* = 0.405, *p* = 5.3*e* − 8), N classification (*r* = 0.326, *p* = 7.8*e* − 7), and M classification (*r* = 0.190, *p* = 0.037), but TXNRD1 level was not significantly correlated with T classification (*r* = 0.178, *p* = 0.052) (Table 4). Taken together, high TXNRD1 level was positively correlated with clinical stage, N classification, and M classification.

### 3.3. Patients with High TXNRD1 Level Have Poor Outcome

**Figure 3:**
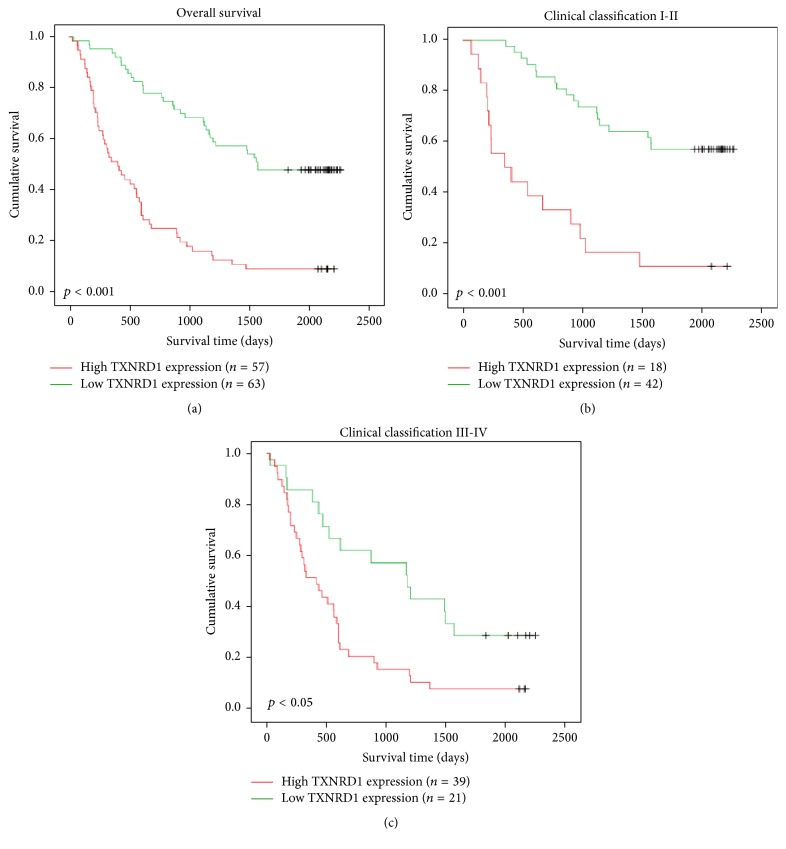
Kaplan-Meier analysis to investigate the prognostic value of TXNRD1 level in overall survival and different clinical stage. (a) All patients. (b) Patients with clinical classification I-II. (c) Patients with clinical classification III-IV.

Using Kaplan-Meier analysis and the log-rank test, we observed that patients with low TXNRD1 levels had longer overall survival time (*p* < 0.001, Figure 3(a)). Subgroup analyses found that patients with high TXNRD1 levels had poor prognosis in the clinical classification I-II (*p* < 0.001) or in the clinical classification III-IV (*p* < 0.05), suggesting TXNRD1 also was an unfavorable prognostic factor for clinical classifications I-II and III-IV (Figures 3(a) and 3(b)). Univariate Cox-regression analysis showed both clinical stage and TXNRD1 level were the significant poor prognostic factors (*p* = 5.5*e* − 9 and *p* = 8.5*e* − 8, resp.), and multivariate Cox-regression analysis found clinical stage and TXNRD1 level were independent prognostic factor for HCC patients (*p* = 6.1*e* − 7 and 5.4*e* − 11, resp.) (Table 5). These findings revealed that TXNRD1 was an independent prognostic factor for HCC patients; patients with high TXNRD1 level had shorter survival time than those who had low TXNRD1 level.

## 4. Discussion

In the present study, we investigated whether TXNRD1 could function as a prognostic factor for HCC patients. We first analyzed TXNRD1 expression using public database and found that TXNRD1 was significantly overexpressed in HCC tissues, and patients with high TXNRD1 level had poor outcome. Then, we further determined TXNRD1 expression in HCC cells and clinical specimens; TXNRD1 was overexpressed in HCC cells, and almost all collected specimens were TXNRD1-positive. Statistical analyses revealed that high TXNRD1 expression was positively correlated with clinical stage, N classification, and M classification. The patients who had low TXNRD1 level had longer survival time. Multivariate analysis showed TXNRD1 was an independent prognostic biomarker for HCC patients.

Thioredoxin system comprised by thioredoxin reductase (TrxR/TXNDR), thioredoxin (Trx), and NADPH plays an important role in maintaining intracellular redox homeostasis. There are three TXNDRs: TXNRD1 is located in cytosol, TXNRD2 is located in mitochondria, and TXNRD3 is located in testis [14]. IHC analysis also found TXNRD1 was mainly presented in cytosol, and the role and molecular mechanisms of TXNRD1 in HCC progression will be explored further.

TXNRD1 is involved in protecting from ROS by itself and via its function together with TXN to serve as an electron donor for peroxiredoxins or ribonucleotide reductase involved in DNA replication and repair [14]. TXNRD1 has been demonstrated to be overexpressed in many cancers, and ROS level also increased in many tumors, including HCC [15]. TXNRD1 promotes tumor growth, DNA replication, and tumorigenicity [16, 17], and knockdown of TXNRD1 also increases sensitivity of cancer cells to some chemotherapy drugs [18, 19]; these finding suggest it is a potential target for anticancer agents [20]. In HCC, ROS causes DNA damage and regulates p53, AP1, NF-AT, Nrf2/Maf, and multidrug resistance proteins to promote hepatocarcinogenesis and drug resistance [21]; we conferred that TXNRD1 might induce ROS generation to promote HCC development and drug resistance.

We found TXNRD1 could function as an unfavorable prognostic factor for HCC, while this finding is still to be replicated and to be verified in other patients' population. In summary, we found TXNRD1 was upregulated in HCC tissues and cells, and TXNRD1 level was increased with increasing clinical stage. High TXNRD1 level was positively correlated with clinical stage, N classification, and M classification, and patients with high TXNRD1 level had poor outcome; TXNRD1 was a novel biomarker for the prognosis for HCC patients.

## Figures and Tables

**Table 1:** Clinicopathological characteristics of HCC patients' samples.

	Number of cases (%)
Gender	
Male	88 (73.3%)
Female	32 (26.7%)
Age (years)	
≥45	76 (63.3%)
<45	44 (36.7%)
Clinical stage	
I	30 (25.0%)
II	30 (25.0%)
III	34 (28.3%)
IV	26 (21.7%)
T classification	
T1	39 (32.5%)
T2	33 (27.5%)
T3	18 (15.0%)
T4	30 (25.0%)
N classification	
N0	98 (81.7%)
N1	22 (18.3%)
M classification	
Yes	7 (5.8%)
No	113 (94.2%)

**Table 2:** The expression of TXNRD1 in HCC.

Expression of TXNRD1	
Negative	7 (5.8%)
Positive	113 (94.2%)
Low expression	63 (52.5%)
High expression	57 (47.5%)

**Table 3:** Correlation between TXNRD1 expression and clinicopathological characteristics of HCC.

Characteristics	TXNRD1		Chi-square test *p* value	Fisher's exact test *p* value
	Low number of or no cases	High number of cases		
Gender				
Male	44	44	0.363	0.412
Female	19	13		
Age (years)				
≥45	40	36	0.970	1.000
<45	23	21		
Clinical stage				
I	23	7	3.5*e* − 5	3.5*e* − 5
II	19	11		
III	16	18		
IV	5	21		
T classification				
T1	24	15	0.265	0.263
T2	19	14		
T3	8	10		
T4	12	18		
N classification				
N0	59	39	4.4*e* − 4	0.001
N1	4	18		
M classification				
No	62	51	0.037	0.052
Yes	1	6		

**Table 4:** Spearman correlation analysis between TXNRD1 and clinical pathologic factors.

Variables	TXNRD1 expression level	
	Spearman correlation	*p* value
Clinical stage	0.405	5.3*e* − 8
T classification	0.178	0.052
N classification	0.326	7.8*e* − 7
M classification	0.190	0.037

**Table 5:** Univariate and multivariate analyses of various prognostic parameters in patients with HCC Cox-regression analysis.

	Univariate analysis			Multivariate analysis		
	Number of patients	*p*	Regression coefficient (SE)	*p*	Relative risk	95% confidence interval
Clinical stage						
I	30	5.5*e* − 9	1.807 (0.111)	6.1*e* − 7	0.396	1.183–1.867
II	30					
III	34					
IV	26					
Expression of TXNRD1						
Low expression	63	8.5*e* − 8	3.970 (0.230)	5.4*e* − 11	1.071	1.792–4.755
High expression	57					
